# Biogenic selenium extract-mediated and total *Convolvulus oxyphyllus* extracts suppress IL6 and COX2 expression: insights from LC–MS metabolite profiling and molecular docking

**DOI:** 10.1038/s41598-026-49047-4

**Published:** 2026-05-13

**Authors:** Mohamed D. Abd El-Halim, Nawal H. Mohamed, Reham M. El-Meligy, Rehab A. Lotfy

**Affiliations:** 1 Phytochemistry unit, Department of Medicinal and Aromatic Plant, Desert Research Centre, Cairo, Egypt; 2 Natural product unit, Department of Medicinal and Aromatic Plant, Desert Research Centre, Cairo, Egypt

**Keywords:** Flavonoids, *Convolvulus oxyphyllus*, Anti-inflammatory, IL-6, COX-2, Molecular docking, Biochemistry, Computational biology and bioinformatics, Drug discovery, Plant sciences

## Abstract

Inflammation plays a crucial role in many chronic diseases with IL-6 and COX-2 being key mediators. The current article explored the anti-inflammatory potential of *Convolvulus oxyphyllus* extracts, combining phytochemical profiling, molecular docking, and gene expression analysis to elucidate their mechanisms of action. Total alcohol extract and extract-mediated Se-NPs of *C. oxyphyllus* were evaluated for their ability to modulate IL6 and COX2 expression using quantitative real-time PCR (qRT-PCR). LC–MS investigation was implemented to identify major phytoconstituents, followed by molecular docking to assess the binding interactions of selected most abundant flavonoid compounds (quercetin, daidzein-8-C-glucoside, kaempferol-7-neohesperidoside, and eriodictyol-7-O-neohesperidoside) with COX-2 (PDB ID: 8ET0) and IL-6 (PDB ID: 4N19). Both extract-mediated Se-NPs and total alcohol extract significantly down-regulated IL6 and COX2 expression (69% and 73%, respectively), comparable to Celecoxib. LC–MS profiling revealed the presence of flavonoids as dominant metabolites, alkaloids, coumarins and other constituents. Molecular docking demonstrated strong ligand–target interactions, with eriodictyol-7-O-neohesperidoside showing the highest binding affinity for COX-2 (− 10.2 kcal/mol) and kaempferol-7-neohesperidoside for IL-6 (− 7.4 kcal/mol), surpassing or matching the standard drug Celecoxib. These outcomes emphasize the therapeutic potential of *C. oxyphyllus* and its constituent phytochemicals as natural anti-inflammatory agents warranting further in vivo validation.

## Introduction

Medicinal plants constitute a major reservoir of bioactive compounds and continue to serve as a cornerstone for the discovery of novel therapeutic agents. Extensive phytochemical and pharmacological investigations have been conducted to evaluate their safety, efficacy, and mechanistic potential. Numerous plant families exhibit broad-spectrum biological activities, including anti-inflammatory, antidiabetic, antitumor, antiviral, and antimicrobial effects.

The genus *Convolvulus* is particularly notable for its rich phytochemical composition, including flavonoids, alkaloids, and phenolic compounds^1^. These constituents are widely recognized for their diverse pharmacological properties, especially their ability to modulate inflammatory signaling pathways^2^. Moreover, species within this genus have demonstrated significant antioxidant, antiulcer, and anti-inflammatory activities^3^ and have been traditionally employed in the management of respiratory conditions such as asthma and cough^4^.

Inflammation is a fundamental defense mechanism essential for host protection and tissue repair; however, when persistent or dysregulated, it contributes to the pathogenesis of numerous chronic diseases, including cardiovascular disorders, neurodegenerative conditions, and cancer^5^. Central inflammatory mediators, such as cyclooxygenase-2 (COX-2) and interleukin-6 (IL-6), play pivotal roles in propagating inflammatory cascades, and their sustained overexpression is closely associated with tissue damage and chronic pathological states^6,7^. Chronic low-grade inflammation is also recognized as a key contributor to cardiometabolic risk and age-related functional decline^8,9^.

Importantly, inflammation represents a modifiable biological target. Exercise-based interventions have been shown to attenuate inflammatory burden and improve health outcomes; for example, intermittent walking has been reported to improve cardiometabolic risk factors in postmenopausal women^10^, while resistance training enhances muscle strength and functional performance in older adults^11^. Collectively, these findings underscore the therapeutic relevance of targeting inflammatory pathways to promote cardiometabolic and functional health.

Tissue injury is a well-established trigger of inflammatory responses. Epidemiological studies in police cadets have demonstrated a high incidence of training-related musculoskeletal injuries^12^. Furthermore, mechanical overload and inadequate recovery may exacerbate tissue damage and prolong inflammatory responses^13^, emphasizing tissue injury as a modifiable contributor to overall inflammatory burden.

Nonsteroidal anti-inflammatory drugs (NSAIDs) and selective COX-2 inhibitors, such as celecoxib, exert their anti-inflammatory effects primarily by inhibiting prostaglandin synthesis and modulating cytokine signaling pathways. However, their long-term use is associated with significant adverse effects, including gastrointestinal, cardiovascular, and renal toxicity^14^. These limitations have stimulated increasing interest in the identification and development of natural anti-inflammatory agents, which may serve as promising alternatives or complementary therapies with potentially reduced side effects^15^.

Advances in analytical and computational methodologies now enable comprehensive characterization of plant-derived bioactive compounds. Liquid chromatography–mass spectrometry (LC–MS) is a powerful analytical technique for the precise identification and profiling of phytochemical constituents in complex plant extracts^16^. In parallel, molecular docking has emerged as a valuable computational approach that complements experimental analyses by predicting ligand–protein interactions. Specifically, it allows for the assessment of how phytoconstituents may bind to and potentially modulate key inflammatory targets such as COX-2 and IL-6, thereby providing mechanistic insight^17,18^.

Among plant-derived compounds, flavonoids are particularly noteworthy due to their broad spectrum of biological activities, including antioxidant, anti-inflammatory, and analgesic effects^19–21^. Extensive reviews have highlighted the capacity of flavonoids, such as quercetin, to regulate multiple inflammatory signaling pathways and modulate immune responses^22^.

*Convolvulus oxyphyllus* (Convolvulaceae) is a relatively understudied medicinal plant traditionally used for its analgesic, anti-inflammatory, and antioxidant properties. Despite its ethnopharmacological relevance, the molecular mechanisms underlying its anti-inflammatory effects remain largely unexplored, representing a clear research gap. Moreover, the bioactive constituents of *C. oxyphyllus* may exhibit limited stability and bioavailability, potentially restricting their therapeutic efficacy.

To address these limitations, selenium extract-mediated selenium nanoparticles (Se-NPs) were employed as a nanoformulation strategy to enhance bioavailability and potentiate the activity of plant-derived compounds. Se-NPs are known to exert antioxidant and immunomodulatory effects and to modulate key inflammatory mediators, including interleukin-6 (IL-6) and cyclooxygenase-2 (COX-2). Compared with bulk selenium, Se-NPs demonstrate improved biocompatibility and therapeutic performance. Thus, combining *C. oxyphyllus* extract with Se-NPs represents a novel, mechanism-oriented approach to amplify anti-inflammatory activity while targeting central inflammatory pathways^23–25^.

In addition, molecular docking serves as a valuable in silico tool for predicting interactions between phytoconstituents and inflammatory targets, thereby providing mechanistic insight prior to extensive experimental validation. The integration of nanoformulation with computational modeling supports the central hypothesis that Se-NPs conjugated with *C. oxyphyllus* extract may offer an enhanced, mechanism-driven anti-inflammatory intervention.

Accordingly, this study investigated the anti-inflammatory potential of total alcoholic extract and extract-mediated Se-NPs of *C. oxyphyllus* using an integrated experimental–computational framework. Gene expression analysis was performed to assess the modulation of IL6 and COX2, while LC–MS profiling enabled identification of major phytoconstituents potentially responsible for these effects. Molecular docking simulations were subsequently conducted to evaluate the binding affinities and interaction modes of selected compounds with COX-2 and IL-6. This comprehensive approach aimed to elucidate the molecular basis of the anti-inflammatory activity of *C. oxyphyllus*, bridging phytochemical characterization with mechanistic and computational evidence.

## Materials and methods

### Plant material

The plant’s aerial parts were gathered in April 2024 from South Sinai (Egypt), plant sample was identified and authenticated by Dr. Ahmad el khouly at DRC Herbarium, Desert Research Center. And a comparison to flora of Egypt plant description (Boulos 2000)^26^. Voucher a specimen of the plant was deposited in the herbarium of Desert Research Center with the code number (DRC 20/850). Plant collection and experimental protocol were achieved after permission from the “Desert Research Center, Cairo, Egypt”. Experimental research and field studies on wild plant, including the collection of plant material, comply with relevant institutional, national, and international guidelines and legislation. The plant material was air-dried in a shaded area, and ground into powder.

### Extraction

In 3 L conical flask, 350 gm from last prepared plant powder was macerated in 1.5 L of 70% ethanol for two days at room temperature. Under the same circumstances, this extraction process was carried out three times. The resulting ethanol extracts were combined, fitered through filter paper Whatman No. 1, then evaporated at 40 °C under decreased pressure in a rotavapor. After thorough drying, 40 g of solid extract was obtained from the crude alcohol extract.

### LC/Mass/Mass analysis of the alcohol extract

#### Instrument

The analysis of the sample was carried out by using LC-ESI-MS/MS (liquid chromatography–electrospray ionization–tandem mass spectrometry) with an ExionLC AC system (USA) for isolation and SCIEX Triple Quad 5500 + Mass/Mass system (Singapore) for detection it was connected with ESI (electrospray ionization).For identification of compounds Using Mass-Dial version 4.70 with ResPect library. The detected compounds identifications were assigned using MS-DIAL (Mass Spectrometry Data Independent AnaLysis), version 4.70 (available at: https://systemsomicslab.github.io/compms/msdial/main.html) open-source software in combination with ReSpect positive (2737 records) or ReSpect negative (1573 records) databases were used as the reference database.^27^.

#### Negative ionization mode

The isolation was carried out with a Ascentis^®^ Express 90 Å Column is C18 (2.1 × 150 mm, 2.7 μm). The contents of mobile phases were two eluents A: 5 mM ammonium formate pH 8; B: acetonitrile (LC grade). From 0 to 1 min the mobile phase started at 5% B, from 1 to 20 min increased from 5 to 100% B, from 20 to 25 min was held at 100% B, at 25.01 back to 5%, at 25.01–30 min mobile phase stays at 5% from. The rate of flow was 0.3 ml/min and the volume of injection was 5 µl.

Analyses were performed in negative electrospray ionization (ESI) mode using an EMS-IDA-EPI workflow. For MS¹, the scan range was set from 100 to 1000 Da with the following source parameters: curtain gas, 25 psi; IonSpray voltage, − 4500 V; source temperature, 500 °C; ion source gases 1 and 2, 45 psi. For MS², the scan range was 50 to 1000 Da with a declustering potential of − 80 V and a collision energy of − 35 eV.

Mass/Mass analyses for positive ionization mode were performed using a scan (EMS-IDA-EPI) from 100 to 1000 Da for MS1 with the following source parameters: curtain gas: 25 psi; IonSpray voltage: 5500; source temperature: 500 °C; ion source gases 1 and 2 were 45 psi and scan range was 50 to 1000 Da for MS2 with a declustering potential: 80 V and a collision energy: 35 eV.

#### Green synthesis of selenium extract-mediated Se-NPs (Se-NPs) using *Convolvulus oxphylls* extract

At room temperature, 0.2 g of *C. oxyphyllus* crude alcohol extract was dissolved in 20 milliliters of 70% methanol using a magnetic stirrer. Following full dissolution, 100 ml of the final volume was diluted with distilled water to reach a 2000 ppm extract concentration. In order to create Se-NPs from *C. oxyphyllus* aqueous extract^28^, procedures were modified to optimize the pH, temperature, incubation duration, and precursor concentration. 0.2 g of sodium selenite (Na₂SeO₃) was dissolved in 50 mL of room temperature deionized water and then added to 10 ml of *C. oxyphyllus* extract (filtrate) to create an environmentally friendly synthesis of Se-NPs. To create in-situ Se-NPs at pH 6.2, the mixture was heated to 65 °C for 1 h while being constantly agitated. Following the addition of 200 µL of 40 mM ascorbic acid a secondary reducing agent/catalyst to improve reaction yield and stabilize the particles, while the *C. oxyphylls* extract served as the primary green capping and stabilizing agent, ruby-red Se-NPs were produced, as previously reported^29,30^. Five milliliters of 1% NaOH were added to the solution dropwise so as to bring the pH down to a basic environment at pH 10. Ultimately, the reaction mixtures were supplemented with deionized water to achieve a 2000 ppm concentration. To improve nanoparticle dispersion and avoid agglomeration, the mixture was ultrasonically treated (sonicated) for 30 min at 35 °C after being incubated at room temperature for the entire night. Centrifugation was used to gather the resultant Se-NPs, which were then routinely cleaned with distilled water and kept at 4 °C until used.

### Characterization of Se-NPs

#### Transmission electron microscopy (TEM)

The morphology, size, and distribution of synthesized Se-NPs were examined using a JEOL JEM-2100 F transmission electron microscope (JEOL Ltd., Tokyo) at 200 kV. Samples were prepared by placing a drop of diluted Se-NP suspension onto carbon-coated copper grids (300 mesh) and air-dried. Images were recorded with a Gatan Orius SC1000 CCD camera (Gatan Inc., Pleasanton, CA). Particle size distribution was determined by measuring over 200 particles from multiple images using ImageJ (version IJ 1.54; U.S. National Institutes of Health, Bethesda, Maryland, USA available at: https://imagej.net/ij/download.html).

#### Dynamic light scattering (DLS)

The hydrodynamic diameter, polydispersity index (PDI) of Se-NPs were determined using a Malvern Zetasizer Nano ZS (Malvern Panalytical, UK) with a 633 nm He-Ne laser at 25 °C and a 173° scattering angle. Deionized water was used to dilute the DLS samples before they were passed through 0.22 μm syringe filters. Every measurement was taken three times and averaged.

#### X-ray diffraction (XRD)

A Rigaku SmartLab X-ray diffractometer (Rigaku, Tokyo) using Cu Kα radiation (λ = 1.5406 Å) at 40 kV and 30 mA was used to examine the crystalline structure and phase of Se-NPs. With a 0.02° step size and a 2°/min scan speed, XRD patterns were captured between 10° and 80° 2θ. Concentrated Se-NP suspensions were drop-cast onto silicon zero-background holders to create the samples, which were then allowed to air dry. The Debye-Scherrer equation, D = 0.9λ/βcosθ, was used to determine the crystallite size.

Where D is the crystallite size (nm), λ is the X-ray wavelength (1.5406 Å), β is the full width at half maximum (FWHM) of the diffraction peak (in radians), and θ is the Bragg’s angle.130.

## Anti-inflammatory activity

### Effect on mRNA expression of inflammatory genes (IL6 and COX2) using one-step real-time quantitative RT-PCR

#### Cell treatment and sample preparation

RAW264.7 murine macrophage cells (Vacsera Cell Bank, Giza, Egypt) were used; cells were cultured under standard conditions (37 °C, 5% CO₂) in Dulbecco’s Modified Eagle Medium (DMEM) supplemented with 10% fetal bovine serum (FBS) and 1% penicillin–streptomycin. To establish an in vitro inflammatory model, cells were seeded into 6-well plates and allowed to adhere overnight. Inflammation was induced using lipopolysaccharide (LPS, Escherichia coli O111:B4) at a final concentration of 1 µg/mL for 24 h. Cells were pretreated with: *C. oxyphyllus* total alcohol extract, *C. oxyphyllus* nano-extract and Celecoxib (reference anti-inflammatory drug) for 1 h prior to LPS stimulation. Following pretreatment, LPS (1 µg/mL) was added without removing the test samples, and cells were incubated for 24 h. DMSO was used as vehicle control, with a final concentration maintained below 0.1% (v/v) in all treated groups. After 24 h incubation, cell lysates were collected for total RNA extraction.

#### RNA extraction

Total RNA was isolated from cell lysates applying the Qiagen RNeasy Mini Kit (Qiagen, Germany) following the manufacturer’s instructions. RNA purity and concentration were determined spectrophotometrically (A₂₆₀/A₂₈₀ ratio) using a NanoDrop™ spectrophotometer (Thermo Fisher Scientific). Only RNA samples with A₂₆₀/A₂₈₀ ratios between 1.8 and 2.0 were used for downstream analysis to ensure quality and purity.

#### Quantitative real-time PCR (qPCR)

Quantitative one-step real-time PCR was achieved using the iScript™ One-Step RT-PCR Kit with SYBR^®^ Green Master Mix (Bio-Rad, USA) on a Rotor-Gene™ Real-Time PCR System (Qiagen, Germany). Thermal cycling conditions were as follows: reverse transcription at 50 °C for 10 min, initial denaturation at 95 °C for 5 min, followed by 40 cycles of denaturation at 95 °C for 10 s and annealing/extension at 60 °C for 30 s. A melt-curve analysis (65–95 °C) was performed at the end of each run to verify the specificity of amplification.

**Table:** 

Gene	Forward Primer (5′→3′)	Reverse Primer (5′→3′)	Reference
*IL6*	GAGGATACCACTCCCAACAGACC	AAGTGCATCATCGTTGTTCATACA	NCBI RefSeq
*COX2*	TGAGCAACTATTCCAAACCAGC	GCACGTAGTCTTCGATCACTATC	NCBI RefSeq
*GAPDH*	AGGTCGGTGTGAACGGATTTG	TGTAGACCATGTAGTTGAGGTCA	Housekeeping gene

### Data analysis

Cycle threshold (Ct) values were automatically calculated using Rotor-Gene 6000 Series Software, version 1.7 (Build 87) (Qiagen, Hilden, Germany; available at https://rotor-gene.software.informer.com/1.7/#google_vignette). Expression levels of target genes (*IL6* and *COX2*) were normalized to the housekeeping gene (*GAPDH*) and expressed relative to the LPS-stimulated control group using the 2⁻ΔΔCt method, where:

$$\Delta Ct = Ct_{{t\arg et}} - Ct_{{GAPDH}} ,\,\Delta \Delta Ct = \Delta Ct_{{treated}} - \Delta Ct_{{control}}$$

PCR efficiency (E = 1.903) was determined using standard curves and incorporated into fold change calculations to improve accuracy.

### Molecular docking

The three-D structures of the target receptor proteins (cycloxygenae-2 and interleukin-6) were downloaded from the Protein Data Bank (PDB) (https://www.rcsb.org/). Ligands (quercetin, daidzein-8-C-glucoside, kaempferol-7-neohesperidoside and Eeriodictyol-7-O-neohesperidoside) three-dimensional (3D) structures were downloaded from the PubChem database in SDF format and then optimized. Molecular docking was conducted using PyRx Virtual Screening Tool, version 0.9.8 (available at: https://pyrx.sourceforge.io/) with AutoDock Vina (version 1.2.3) as the docking engine to predict the binding interactions of *Convolvulus oxyphyllus* bioactive compounds with inflammatory targets such as COX-2 and IL-6. Protein structures were retrieved from the Protein Data Bank (PDB) and prepared by removing water molecules, co-crystallized ligands, and adding polar hydrogens. Ligand structures were energy-minimized using the Universal Force Field (UFF) and converted to pdbqt format^31–33^. Docking simulations were performed within a grid box encompassing the active site of each protein; the box dimensions and center coordinates were optimized to fully cover the binding pocket. The default scoring function of AutoDock Vina was used to estimate binding affinities (kcal/mol) and predict binding poses. Redocking of the co-crystallized ligands was performed to validate the docking protocol, ensuring that the predicted binding pose closely reproduced the experimentally observed conformation (RMSD < 2 Å). Key docking parameters included exhaustiveness = 8, number of modes = 10, and energy range = 3 kcal/mol. The resulting docking interactions and binding conformations was visualized and analysed using Discovery Studio client, version 2025 (BIOVIA, Dassault Systèmes; available at: https://www.3ds.com/products/biovia/discovery-studio/visualization). It should be emphasized that these results predict binding potential rather than confirm enzymatic inhibition, and further experimental validation is necessary to substantiate the anti-inflammatory activity.

## Results and discussion

### LC/Mass/Mass analysis of *Convolvulus oxyphyllus* alcohol extract

Negative and positive modes of ESI masses were used in the identification of main 54 compounds in *Convolvulus oxyphyllus* alcohol extract. The compounds included flavonoids (28), phenolic acids and their derivatives (7), coumarine derivatives (2), terpens (2), carboxylic acids and their derivatives (5), alkaloids (2), Purines and pyrimidine derivatives (4), Amino acids derivatives (1) and others (3).

Presented data revealed that flavonoid compounds were represented the major abundance. Results also revealed that most abundant flavonoids in *Convolvulus oxyphyllus* were Quercetin (9.92) followed by Kaempferol-7-neohesperidoside (9.89), Daidzein-8-C-glucoside (9.80) and Eriodictyol-7-O-neohesperidoside (8.4) (Table 1).

The detected compounds identifications were assigned using MS-DIAL version 4.70 open-source software in combination with ReSpect positive (2737 records) or ReSpect negative (1573 records) databases were used as the reference database.^27^.

**Table 1:** LC-ESI-Mass/Mass analysis for chemical compounds of *Convolvulus oxyphyllus* ethanol extract.

No	R_t_. min	Area	Adduct ion	Theo m/z	Obs m/z	Error (ppm)	Mass fragmentation (m/z)	Molecular form	Compd name
1-Flavonoids									
A-Aglycones									
1	1.58	3.14	[M-H]^−^	318	316.98	− 3.2	316.98,317.05, 281.09, 279.10, 249.00, 191.05	C_15_H_10_O_8_	Myricetin
2	8.22	9.92	[M + H]^+^	302	303.09	3.6	303.09,284.87,256.90,228.89,164.96,136.99	C_15_H_10_O_7_	Quercetin
3	8.65	3.64	[M-H]^−^	288	287.03	-3.6	287.03,215.20,161.07,135.11	C_15_H_12_O_6_	3’,4’,5,7 tetrahydroxy flavanone
4	8.98	1.76	[M + H]^+^	286	286.87	3.04	286.87,257,164.95,152.96	C_15_H_10_O_6_	4’,5,7-Trihydroxyflavonol
5	9.16	1.63	[M + H]^+^	316	317.03	3.25	317.03,315.1, 269.1, 257.1, 255.03, 246.89, 169.08	C_16_H_12_O_7_	3’-methoxy-4’,5,7-trihydroxyflavonol
6	20.48	1.91	[M + H]^+^	272	273.02	3.75	273.02,109.02, 150.03, 225.22	C_15_H_12_O_5_	Naringenin
7	12.21	3.64	[M-H]^−^	286	285.1	-3.14	285.1, 239.04	C_15_H_10_O_6_	Luteolin
8	13.12	3.40	[M + H]^+^	270	271.08	4	271.08,271.09, 167.02, 152.01, 131.04, 124.01	C_15_H_10_O_5_	Genistein
9	15.06	1.16	[M-H]^−^	270	269.11	− 3.29	269.11,269.0, 254.04, 106.0	C_15_H_10_O_5_	Apigenin
10	21.44	2.23	[M-H]^−^	254	253.20	− 3.14	253.20,253.19,216.90,169.09,147.68	C_15_H_10_O_4_	Daidzein
11	27.21	2.17	[M-H]^−^	284	283.14	-3.02	283.14,283.09, 179,10, 171.09, 146.95, 135.07, 73.03	C_16_H_12_O_5_	Acacetin
B-glycosides									
12	7.99	1.60	[M + H]^+^	450	451.09	2.42	451.09,449.1, 342.9,, 287.0, 276.9, 151.0, 135.04, 117.03	C_21_H_22_O_11_	Okanin-4’-O-glucoside
13	8.07	4.58	[M + H]^+^	610	610.94	1.54	610.94,302.87,286.90,129.08	C_27_H_30_O_16_	Rutin
14	8.22	6.08	[M + H]^+^	464	465.08	2.32	465.08,403.08,302,286.93,218.95,214	C_21_H_20_O_12_	Quercetin-4’-glucoside
15	8.61	9.89	[M-H]^−^	594	592.92	-1.81	592.92,593.18, 570.67, 547.15, 487.2, 447.1, 392.9	C_27_H_30_O_15_	Kaempferol-7-neohesperidoside
16	8.78	8.12	[M + H]^+^	594	594.98	1.64	594.98,473.10,431.12,353.06	C_30_H_26_O_13_	Kaempferol-3-O-(6"”-p-coumaroyl)-glucoside
17	8.84	8.40	[M + H]^+^	596	597.03	1.72	597.03,595.14, 549.29, 501.1, 433.14, 417.2, 298.1, 296.1	C_27_H_32_O_15_	Eriodictyol-7-O-neohesperidoside
18	8.84	2.15	[M + H]^+^	624	625.05	1.68	625.05,477.09, 431.22, 364.82, 331.04, 315.04, 300.04	C_28_H_32_O_16_	Isorhamnetin-3-O-rhamnoglucoside
19	9.16	1.50	[M + H]^+^	448	448.97	2.16	448.97,447.07, 402.90, 383.23, 365.14, 285.03, 255.02	C_21_H_20_O_11_	Luteolin-7-O-glucoside
20	9.24	5.40	[M-H]^−^	448	447.02	− 2.18	447.02,327.01,284.98,255.05, 227.05,161.04	C_21_H_20_O_11_	Kaempferol-3-O-glucoside
21	9.31	1.81	[M- H]^−^	462	461.15	1.83	461.15,461.1, 435.1, 400.8, 392.9, 324.9, 256.9	C_21_H_18_O_12_	Kaempferol-3-Glucuronide
22	10.29	9.35	[M-H]^−^	611	608.94	− 3.3	608.94,302.20,179.10,161.07	C_27_H_31_O_16_	Delphinidin-3-O-(6’’-O-α-rhamnopyranosyl- β -glucopyranoside)
23	12.00	2.17	[M-H]^−^	740	739.15	− 1.14	739.15, 591.02,301.11	C_33_H_40_O_19_	kaempferol-3-O-robinoside-7-O-rhamnoside
24	18.73	2.76	[M + H]^+^	448	449.23	2.7	449.23,447.07, 402.90, 383.23, 365.14, 285.03, 255.02	C_21_H_20_O_11_	Luteolin-7-O-glucoside
25	23.58	2.24	[M + H]^+^	610	611.33	2.18	611.33,537.12,335.09,300.02,259.04,133.06	C_28_H_34_O_15_	Hesperetin-7-O-neohesperidoside
26	23.59	6.61	[M-H]^−^	464	462.91	− 2.34	462.91,446.93,328.95,311.04,283.22,235.10,161.08,135.03	C_21_H_20_O_12_	Isoquercitrin
27	24.41	1.03	[M + H]^+^	592	593.28	2.16	593.28,454.92,386.94,316.94,248.96,10.97	C_28_H_32_O_14_	Acacetin-7-O-rutinoside
28	27.78	9.80	[M-H]^−^	416	415.18	-1.97	415.18,415.1, 319.12, 269.1, 248.9,	C_21_H_20_O_9_	Daidzein-8-C-glucoside
2-phenolic acids and derivatives									
29	1.46	9.84	[M + H]^+^	122	122.95	7.78	122.95,108.06,107.03,104.097,103.03	C_7_H_6_O_2_	Benzoic acid
30	1.51	4.78	[M-H]^−^	154	153.01	− 6.42	153.01, 135.001, 109.03, , 109.02, 67.01	C_7_H_6_O_4_	2,5-dihydroxybenzoic acid
31	1.93	1.96	[M-H]^−^	138	136.93	− 7.75	136.93,117.05,108.01	C_7_H_6_O_3_	Salicylic acid
32	7.00	1.80	[M-H]^−^	354	353.13	− 2.45	353.13,191.06,179.06,135.07	C_16_H_18_O_9_	Chlorogenic acid
33	9.58	1.66	[M-H]^−^	386	385.10	− 2.33	385.10,301.01,270.96,179.12,161.13,135.09	C_17_H_22_O_10_	1-O-β-D-glucopyranosyl sinapate
34	11.49	6.70	[M + H]^+^	208	209	4.80	209,191.07,161.04,144.95,135,116.98	C_11_H_12_O_4_	3,4-Dimethoxy cinnamic acid
35	13.62	3.87	[M + H]^+^	180	181.09	6.61	181.09, 161.04, 135.04, 85.03, 71.01, 59.01	C_9_H_8_O_4_	Caffeic acid
3-coumarins									
36	1.17	4.17	[M-H]^−^	178	177.01	− 5.56	177.01, 163.1, 151.08, 133.0, 119.05, 85.03	C_9_H_6_O_4_	Daphnetin
37	1.71	5.73	[M-H]^−^	192	191.12	− 4.58	191.12,176,148,104.02	C_10_H_8_O_4_	Scopoletin
4-Terpenes									
38	12.46	1.03	[M + H]^+^	296	297.26	4.25	297.26,281.69,268.76,248.8,206.9,173.06,131.09,108.98	C_20_H_40_O	Phytol
39	13.67	1.57	[M-H]^−^	222	221.15	− 3.82	221.15, 206.05, 161.01, 132.98, 105.01	C_15_H_26_O	Farnesol
5- carboxylic (organic acid) and derivatives									
40	1.36	1.30	[M-H]^−^	134	132.98	− 7.61	132.98, 115.08, 71.017	C_4_H_6_O_5_	D- (+)-Malic acid
41	1.51	1.45	[M-H]^−^	116	114.9	− 9.48	114.90,98.10,88.20,60.1	C_5_H_8_O_3_	Ketovaline
42	1.64	1.34	[M-H]^−^	118	116.94	− 8.98	116.94, 99.91, 73.02	C_4_H_6_O_4_	Succinic acid
43	28.72	2.23	[M-H]^−^	174	173.04	− 5.51	173.04,154.98,137.03,119.03	C_7_H_10_O_5_	Shikimic acid
44	28.65	2.52	[M + H]^+^	223	224.02	4.57	224.02,164.99,149.02,121.04,118.99,103.01	C_12_H_17_ NO_3_	Cerulenin
6-alkaloids									
45	1.79	5.33	[M + H] ^+^	139	140.07	7.69	140.07,125.01,122.06,112.01,108	C_8_H_13_NO	Tropinone
46	8.38	1.04	[M + H]^+^	162	163.13	6.97	163.13,145.09,134.97,106.99	C_10_H_14_N_2_	Nicotine
7-Purines and pyrimidine derivatives									
47	1.44	2.31	[M-H]^−^	152	150.96	− 6.84	150.96,136.01, 108.02, 95.01, 92.02, 91.01,	C_5_H_4_N_4_O_2_	Xanthine
48	2.11	4.96	[M + H]^+^	364	365.07	2.93	365.07,202.96,184.97,167.04	C_10_H_14_N_4_O_9_P	Xanthosine-5’-monophosphate
49	7.06	1.51	[M-H]^−^	166	164.96	− 6.26	164.96,139.10,111.30	C_6_H_6_N_4_O_2_	3-Methylxanthine
50	19.75	2.47	[M-H]^−^	156	155.03	− 6.21	155.03,154.9, 111.04	C_5_H_4_N_2_O_4_	Oroticacid
8-Amino acids derivatives									
51	1.87	2.24	[M + H]^+^	226	227.10	4.86	227.10,209.17,196.02,177.95,166.96,135.09,121.11	C_9_H_14_N_4_O_3_	Carnosine
9- Others									
52	1.93	2.46	[M + H]^+^	201	202.13	5.62	202.13,184.08,166.20,142.12,110.07	C_10_H_7_N_3_S	Thiabendazole
53	2.53	1.01	[M + H]^+^	108	109	9.25	109.00,108.20,107.02	C_6_H_4_O_2_	1,4-Benzoquinone
54	23.83	2.00	[M-H]^−^	406	404.98	− 2.51	404.98, 359.21, 357.13, 297.07, 243.06, 195.06, 153.01	C_20_H_22_O_9_	E-3,4,5’-Trihydroxy-3’- glucopyranosyl stilbene.

### Characterization of Rh-Se-NPs

According to TEM analysis in . 1, the green-synthesized selenium extract-mediated Se-NPs generated with aqueous extract of *C. oxyphyllus* were primarily spherical morphology. The consistent, black circular shapes of the extract-mediated Se-NPs on a lighter backdrop suggested that the selenium ions were effectively reduced and that stable particles were formed. Indicating effective surface capping by *C. oxyphyllus* molecules, minimal aggregation was seen. TEM images displaying the major series of particle size that were between 3.18 and 17 nm. The lack of asymmetrical shapes or sizable clusters bolsters the function of the hydroxyl and carbonyl groups in *C. oxyphyllus* molecules in maintaining the stability of the extract-mediated Se-NPs surface. DLS analysis in . 2 showed that Se-NPs had an intensity-weighted mean hydrodynamic diameter of 88.6 nm. The polydispersity index (PDI) was 0.09, indicating a highly monodisperse extract-mediated Se-NPs population with a unimodal size distribution and narrow peak. X-ray diffraction analysis (XRD), shown in . 3, confirmed the crystalline nature and phase purity of Se-NPs, showing distinct peaks at 2θ values of 23.51°, 29.70°,41.32^o^, 43.65^o^, 45.36^o^ and 51.71°corresponding to the Miller indices assignments (-100), (-10-1), (-210), (-102), (-21-1), and (-20-1) confirms the successful formation of trigonal selenium (t-Se) with hexagonal crystal structure. The intense peak at 29.70° ((-10-1) plane) indicates a preferred crystallographic orientation during biosynthesis. Sharp, well-defined peaks verify high crystallinity. The excellent match between experimental peak positions and reference data (COD 9012501)^34^ validates the hexagonal crystal structure with space group P 3■ 2 1. The lattice parameters calculated from the peak positions (a = 4.366 Å, c = 4.954 Å) are in excellent agreement with literature values for trigonal selenium, demonstrating the structural integrity of the synthesized extract-mediated Se-NPs. the significant difference between the TEM core size (17 nm) and the DLS hydrodynamic size (88.6 nm). This discrepancy is likely due to the formation of a dense bio-organic capping layer from the plant extract and a degree of agglomeration in the aqueous dispersion medium, which DLS is highly sensitive.

**Fig. 1:**
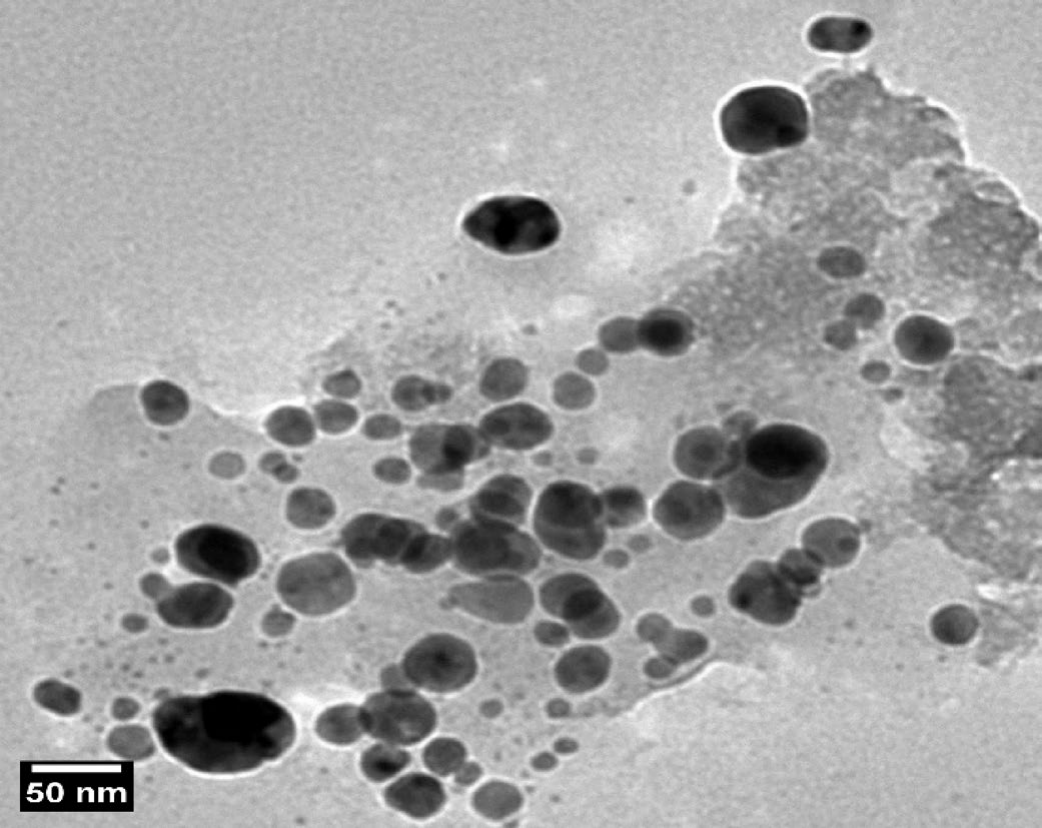
TEM analysis.

**Fig. 2:**
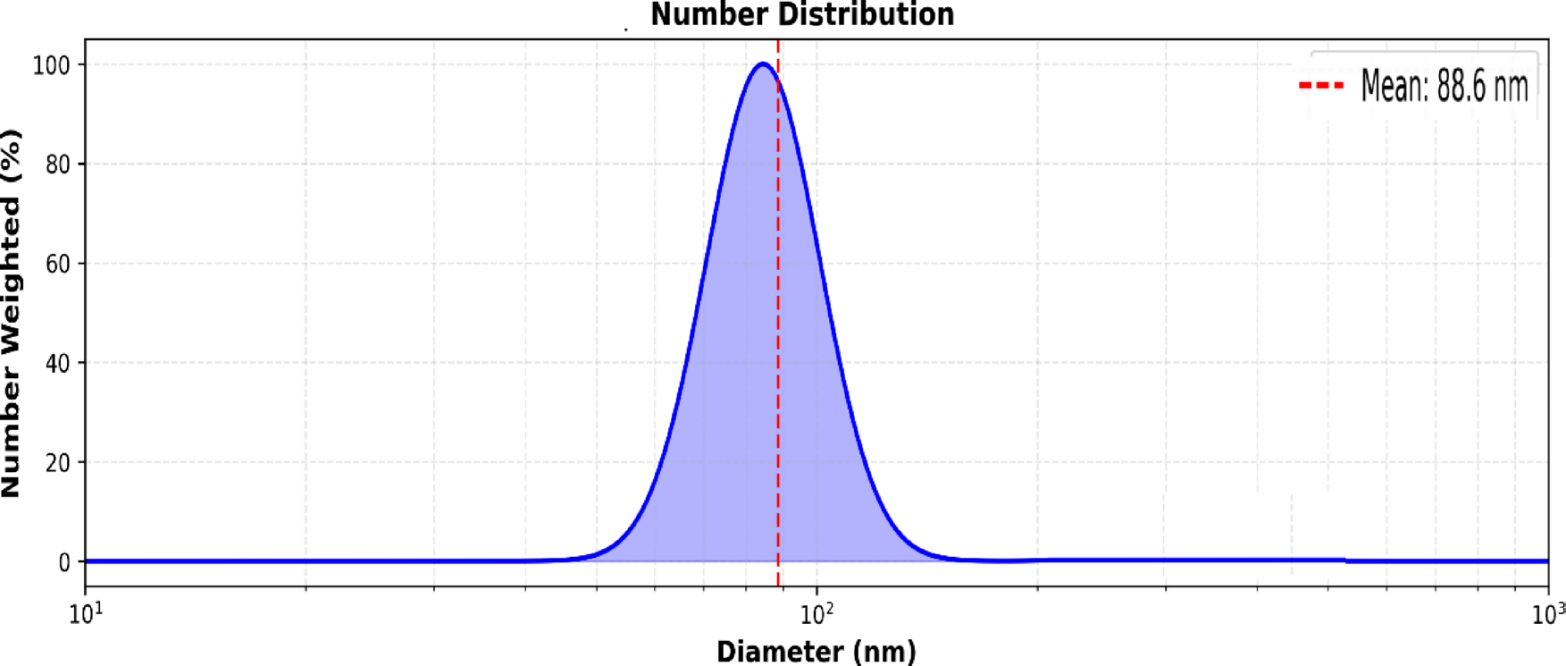
DLS analysis.

**Fig. 3:**
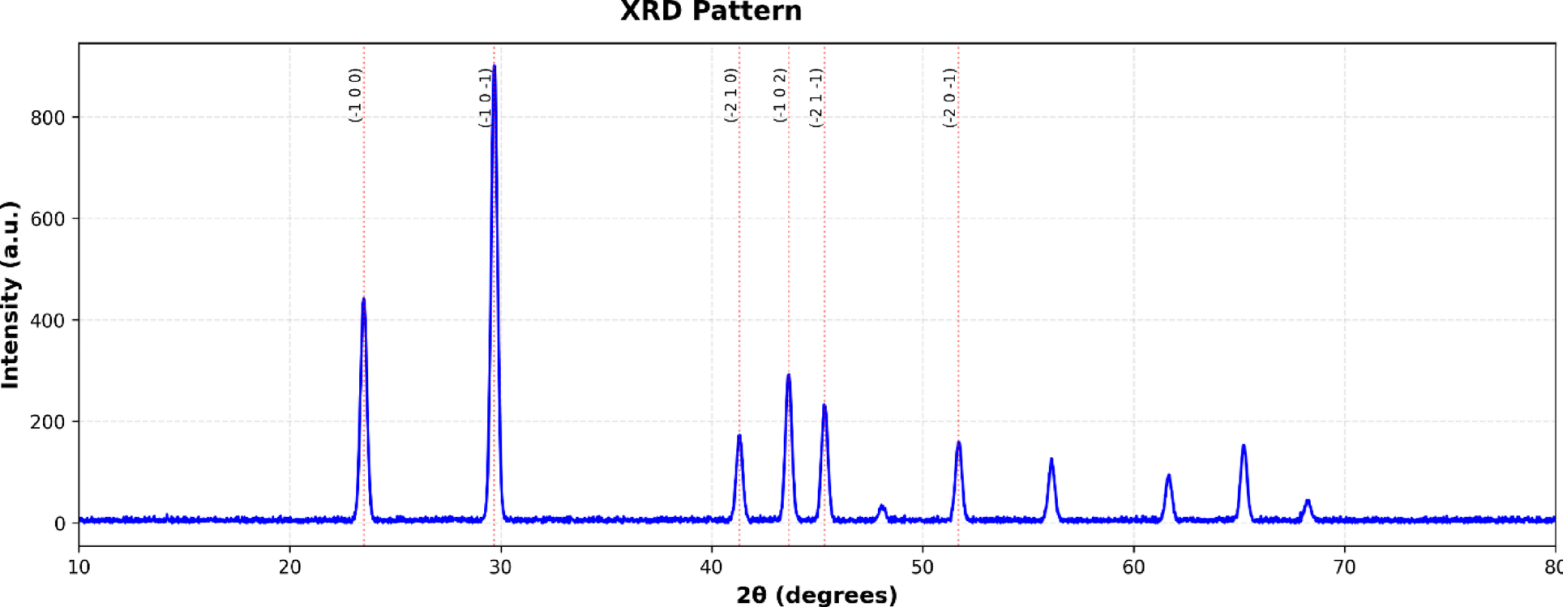
X-ray diffraction analysis (XRD).

## Anti-inflammatory activity

### Effect on mRNA expression of inflammatory genes (IL6 and COX2) using one-step real-time quantitative RT-PCR

Quantitative real-time PCR analysis showed that treatment of RAW264.7 macrophages with *C. oxyphyllus* total alcohol extract, *C. oxyphyllus* extract-mediated Se-NPs, and celecoxib obviously downregulated the mRNA expression of the pro-inflammatory genes IL6 and COX2 compared with the LPS-stimulated control (Table 2). Using GAPDH as the internal reference housekeeping gene, relative quantification by the 2⁻ΔΔCt method revealed that IL6 expression was declined to 0.44 fold in *C. oxyphyllus* total alcohol extract-treated cells, 0.31 fold in *C. oxyphyllus* extract-mediated Se-NPs -treated cells, and 0.28 fold in celecoxib-treated cells. Furthermore, COX2 expression decreased to 0.36 fold, 0.27 fold, and 0.20 fold for *C. oxyphyllus* total alcohol extract, *C. oxyphyllus* extract-mediated Se-NPs and celecoxib-treated cells respectively, relative to LPS-stimulated control (set to 1.0).

**Fig. 4:**
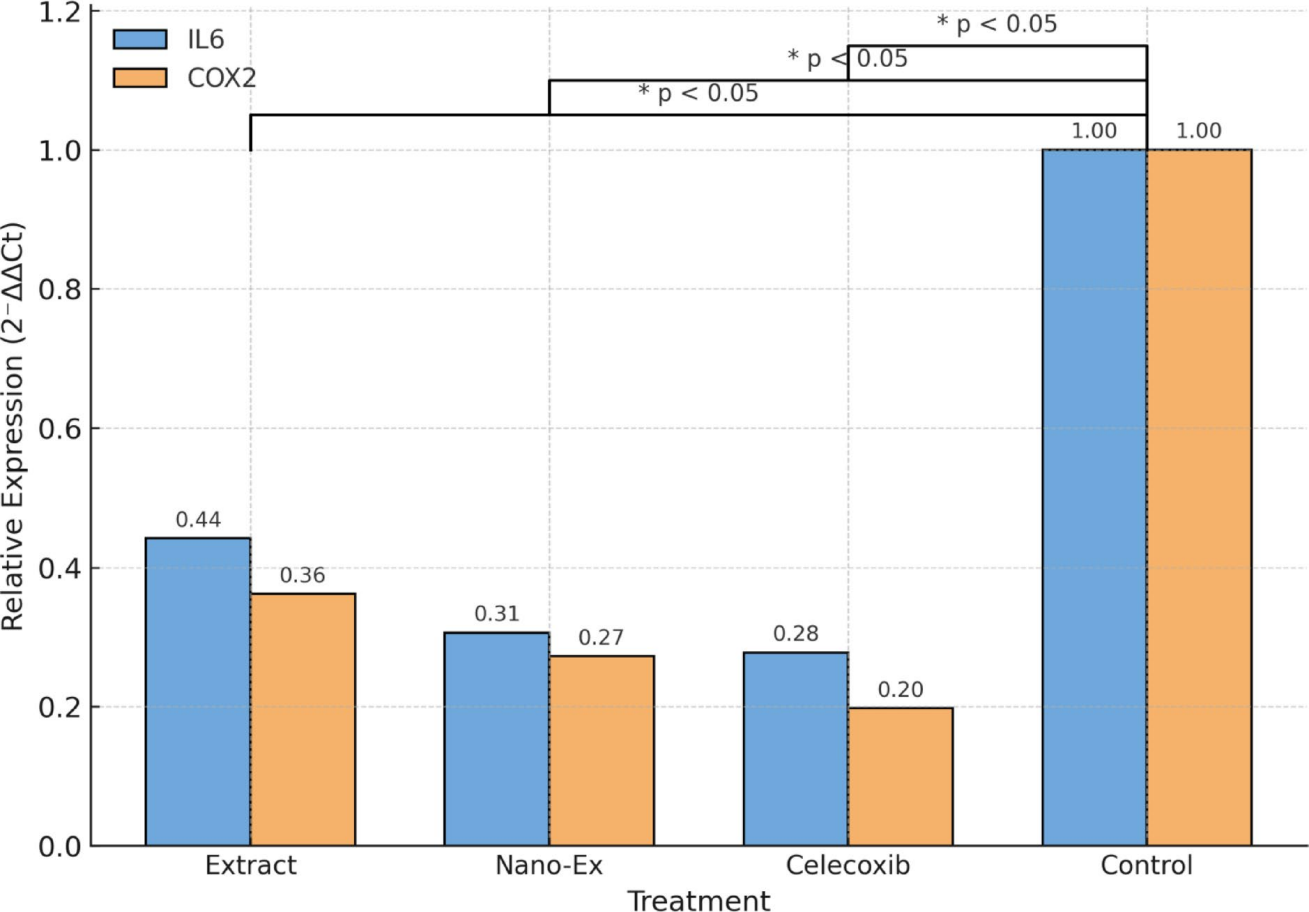
Relative mRNA expression of IL6 and COX2 in LPS-stimulated RAW264.7 macrophages treated with *Convolvulus oxyphyllus* extracts and celecoxib. Expression levels were normalized to GAPDH and calculated using the efficiency-corrected 2⁻ΔΔCt method (E = 1.903).

In addition, results showed that *C. oxyphyllus* extract-mediated Se-NPs exhibited excellent suppression of both IL6 and COX2, indicating enhanced anti-inflammatory efficacy compared to *C. oxyphyllus* total alcohol extract and a response comparable to the standard anti-inflammatory drug celecoxib. These findings suggest that both the *C. oxyphyllus* total alcohol extract and its extract-mediated Se-NPs possess These findings suggest anti-inflammatory potential through transcriptional modulation of inflammatory mediators in macrophages (Fig. 4).

Data are normalized to *GAPDH* and expressed relative to the LPS-stimulated control using the 2⁻ΔΔCt method. PCR efficiency (E) = 1.903 was applied. Lower fold change (< 1) indicates downregulation relative to LPS-stimulated control.

In the present study, treatment of RAW264.7 macrophages with *C. oxyphyllus* total alcohol extract, *C. oxyphyllus* extract-mediated Se-NPs and celecoxib showed significant suppression of both IL6 and COX2 expression, key mediators of inflammation. These findings validate the anti-inflammatory capacity of the plant’s bioactive compounds at the transcriptional level. Similar findings have been reported for other medicinal plants, where phytochemicals could significantly inhibit both cytokine expression and prostaglandin biosynthesis through suppression of COX-2 and IL-6 pathways^35,36^. Likewise, both leaf and fruit extracts of *Terminalia ferdinandiana* showed potent inhibition of COX-2 protein levels in macrophages^37^.

Moreover, the extract-mediated Se-NPs extract achieved up to a 69% reduction in IL6 expression whereas the total alcohol extract reduced it by approximately 56%. These results suggested that nanoscale delivery enhances bioavailability and cellular uptake of active phytoconstituents an effect consistent with other nano phytotherapy studies^38^.

Moreover, nano-formulations designed to target inflammation have been shown to reprogram macrophage phenotype and reduce pro-inflammatory gene expression^39^. By using nano-carriers, the extract’s components are protected and can be delivered more effectively to target sites, leading to enhanced therapeutic effects, thus increasing the activity^40,41^.

Furthermore, the recorded more noticeable suppression of COX2 compared with IL6 (up to 80% reduction for Celecoxib) is consistent with the hierarchical role of COX-2 in prostaglandin-mediated inflammation downstream of IL-6 signaling^7^. Moreover, recent studied indicates that plant-derived polyphenols can modulate both NF-κB and AP-1 transcriptional activity, producing broad suppression of inflammatory mediators^42^.

**Table 2:** Relative mRNA expression of inflammatory genes (IL6 and COX2) in RAW264.7 cells treated with different samples.

Sample	Gene	ΔCt (Control)	ΔCt (Treated)	ΔΔCt	Fold Change (2⁻ΔΔCt)	Expression reduction vs. control
*Convolvulus oxyphyllus* total alcohol extract	IL6	5.12	6.39	1.27	0.44	56%
*Convolvulus oxyphyllus* extract-mediated Se-NPs	IL6	5.12	6.96	1.84	0.31	69%
Celecoxib	IL6	5.12	7.11	1.99	0.28	72%
Control	IL6	5.12	5.12	0.00	1.00	Baseline
*Convolvulus oxyphyllus* total alcohol extract	COX2	5.88	7.46	1.58	0.36	64%
*Convolvulus oxyphyllus* extract-mediated Se-NPs extract	COX2	5.88	7.90	2.02	0.27	73%
Celecoxib	COX2	5.88	8.40	2.52	0.20	80%
Control	COX2	5.88	5.88	0.00	1.00	Baseline

### Molecular docking of *Convolvulus oxyphyllus* constituents targeting cycloxygenae-2 and interleukin-6

Molecular docking is a valuable computational approach in natural product research, enabling prediction of ligand–protein interactions and estimation of binding affinity. The calculated binding energy reflects the predicted stability of the ligand–target complex, with more negative values generally indicating stronger interactions¹⁷˒¹⁸. However, docking results represent theoretical models based on static protein structures and should be interpreted cautiously.

Interleukin-6 (IL-6) is a multifunctional cytokine that mediates inflammation through protein–protein interactions with its receptor complex (IL-6R/gp130), activating downstream JAK/STAT signaling⁹. Because IL-6 is not a classical enzyme with a well-defined catalytic pocket, docking predictions may not directly translate into functional inhibition. While predicted binding poses can identify potential interaction sites and guide compound prioritization, they do not confirm suppression of IL-6 signaling. Therefore, experimental validation through cytokine quantification, receptor-binding assays, and cell-based functional studies remains essential⁹.

**Fig. 5:**
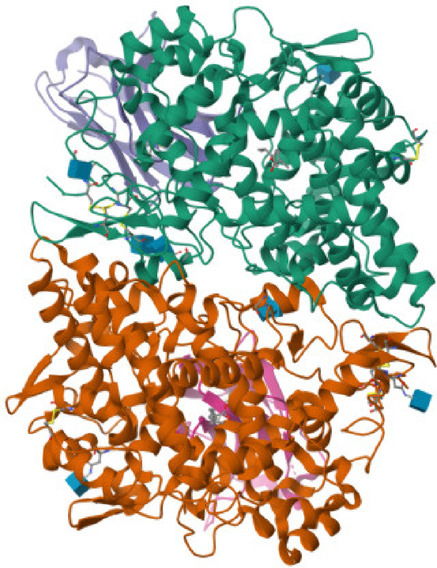
Cycloxygenae-2 (8ET0).

**Fig. 6:**
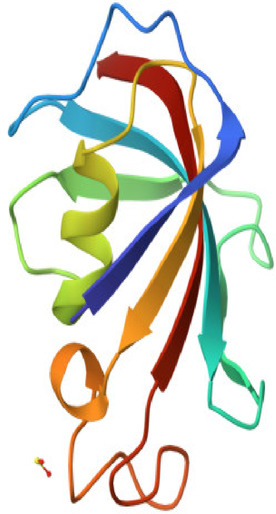
Interleukin-6 (4N19).

**Fig. 7:**
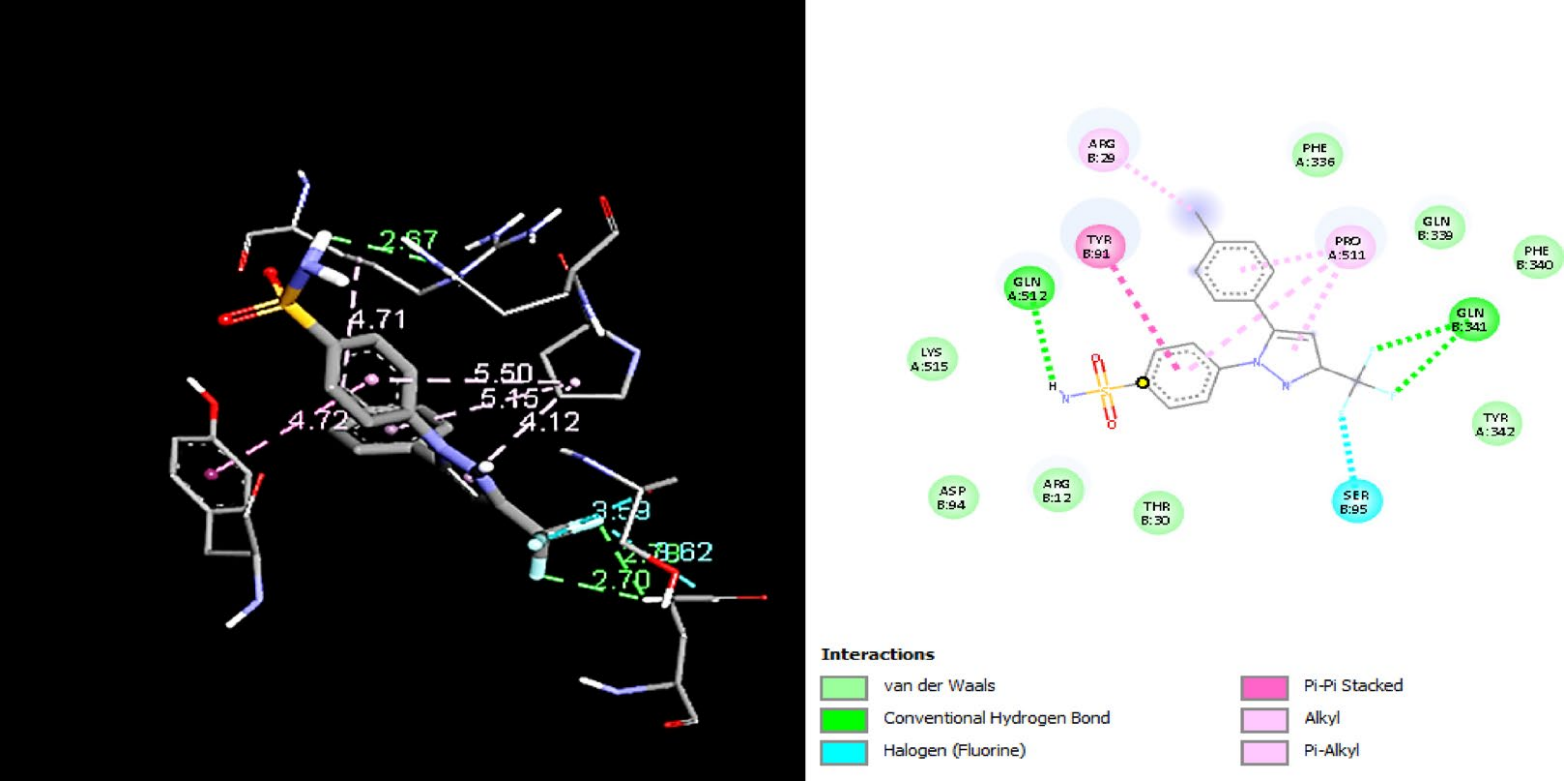
3D and 2D interactions of Celecoxib within cycloxygenae-2 active site.

**Fig. 8:**
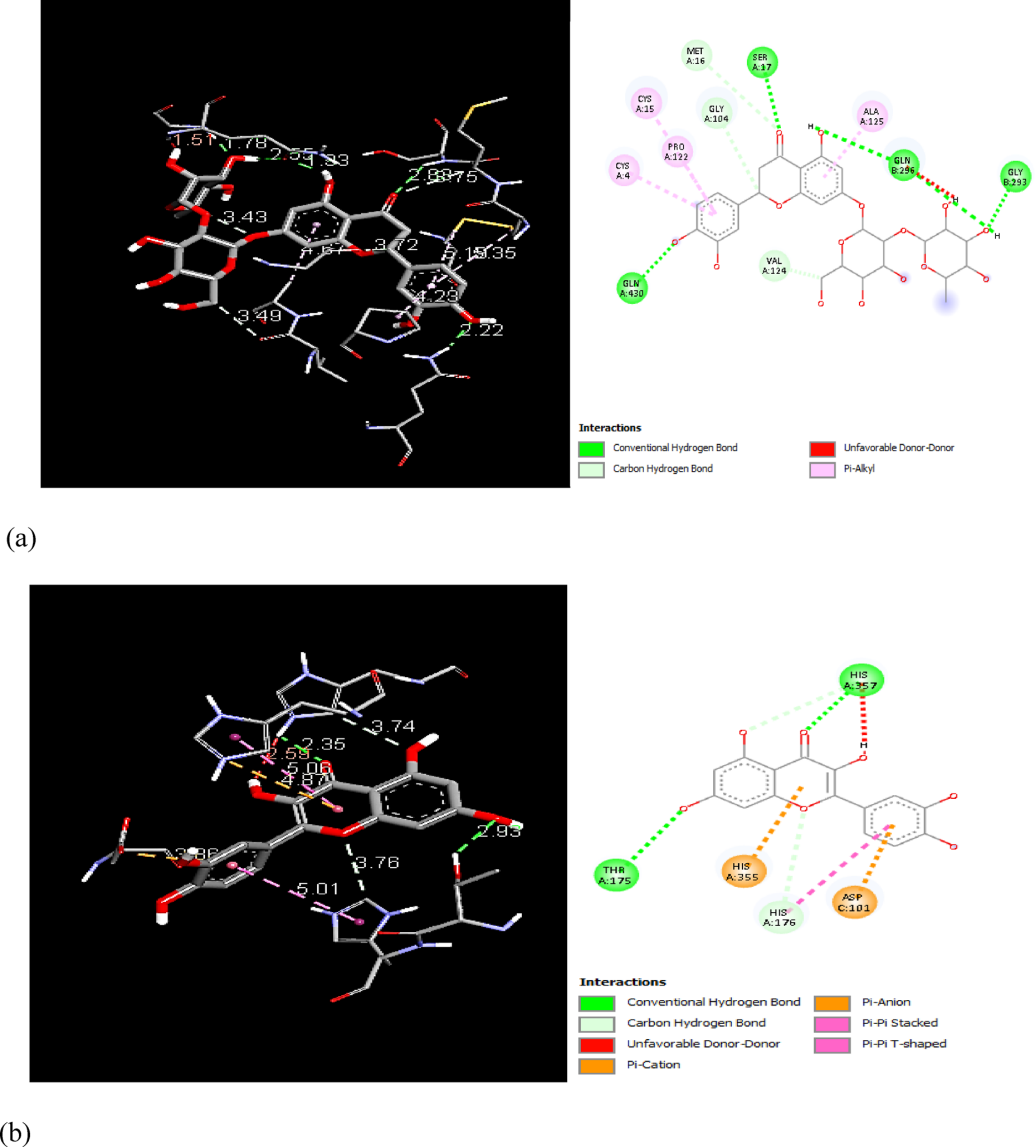

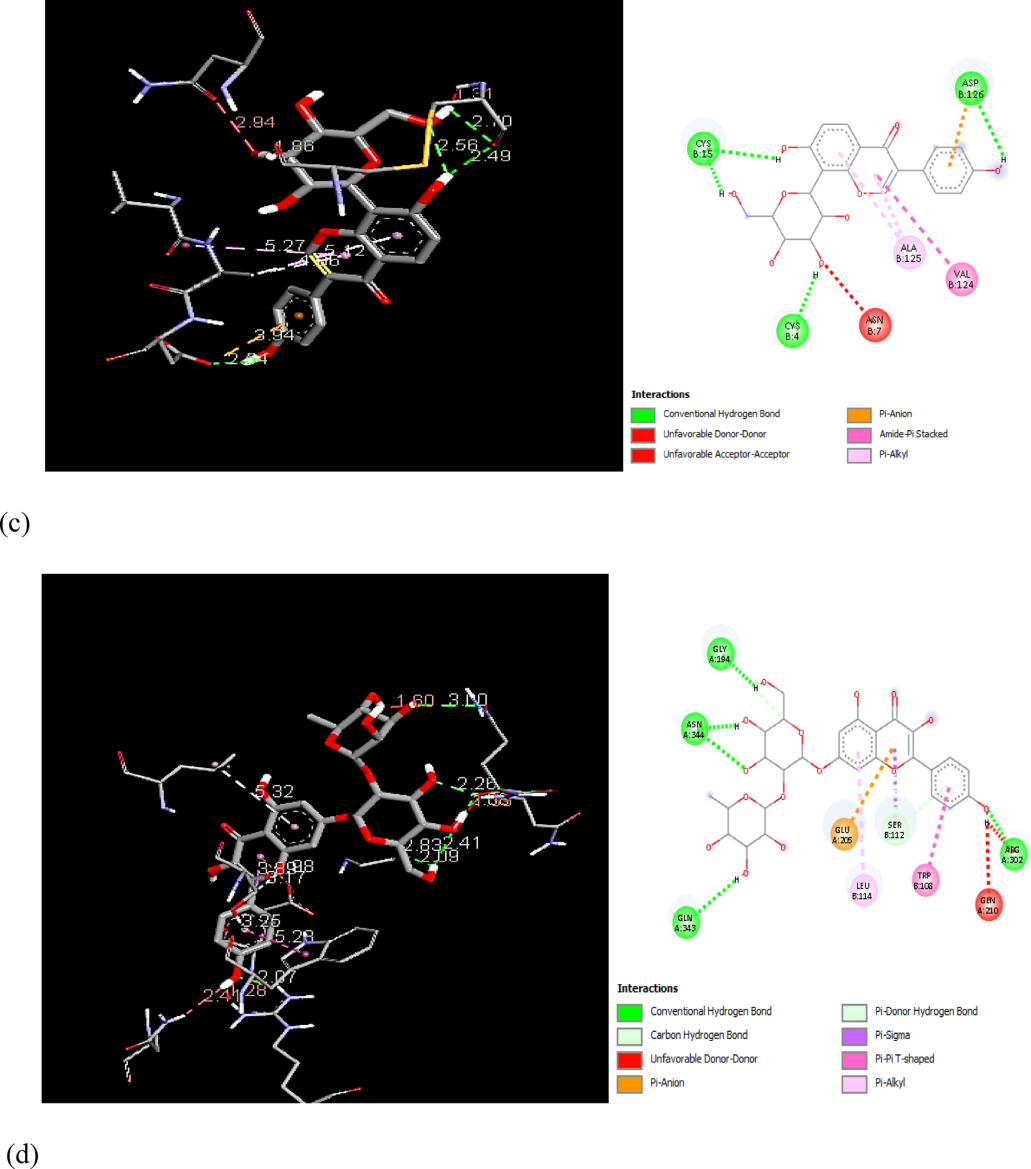
3D and 2D interactions of the isolated compounds from *Convolvulus oxyphyllus Boiss* within cycloxygenae-2 active site. (**a**) Eeriodictyol-7-O-neohesperidoside, (**b**) Quercetin, (**c**) Daidzein-8-C-glucoside, and (**d**) Kaempferol-7-neohesperidoside.

**Fig. 9:**
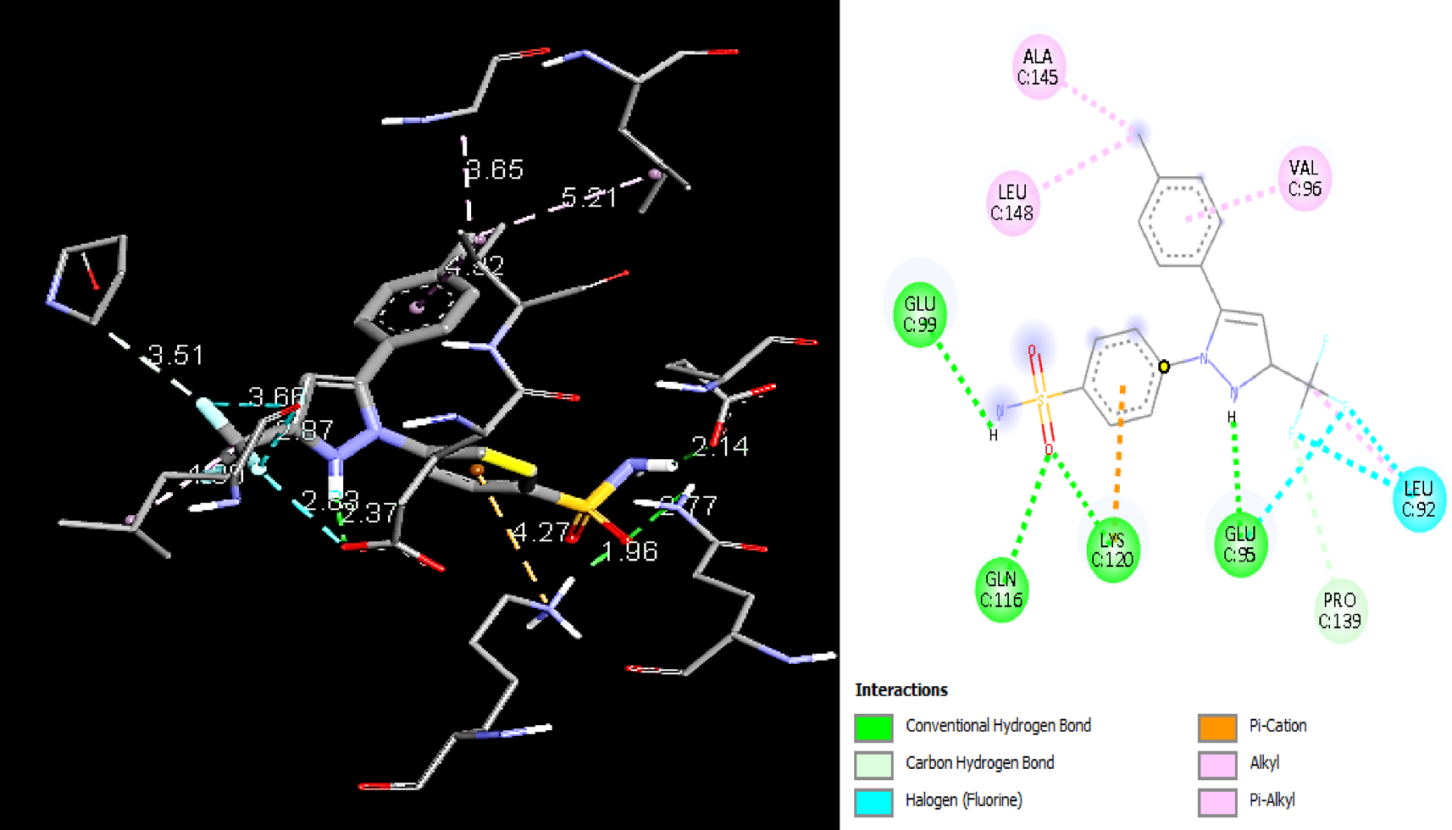
3D and 2D interactions of Celecoxib within interleukin-6 active site.

**Fig. 10:**
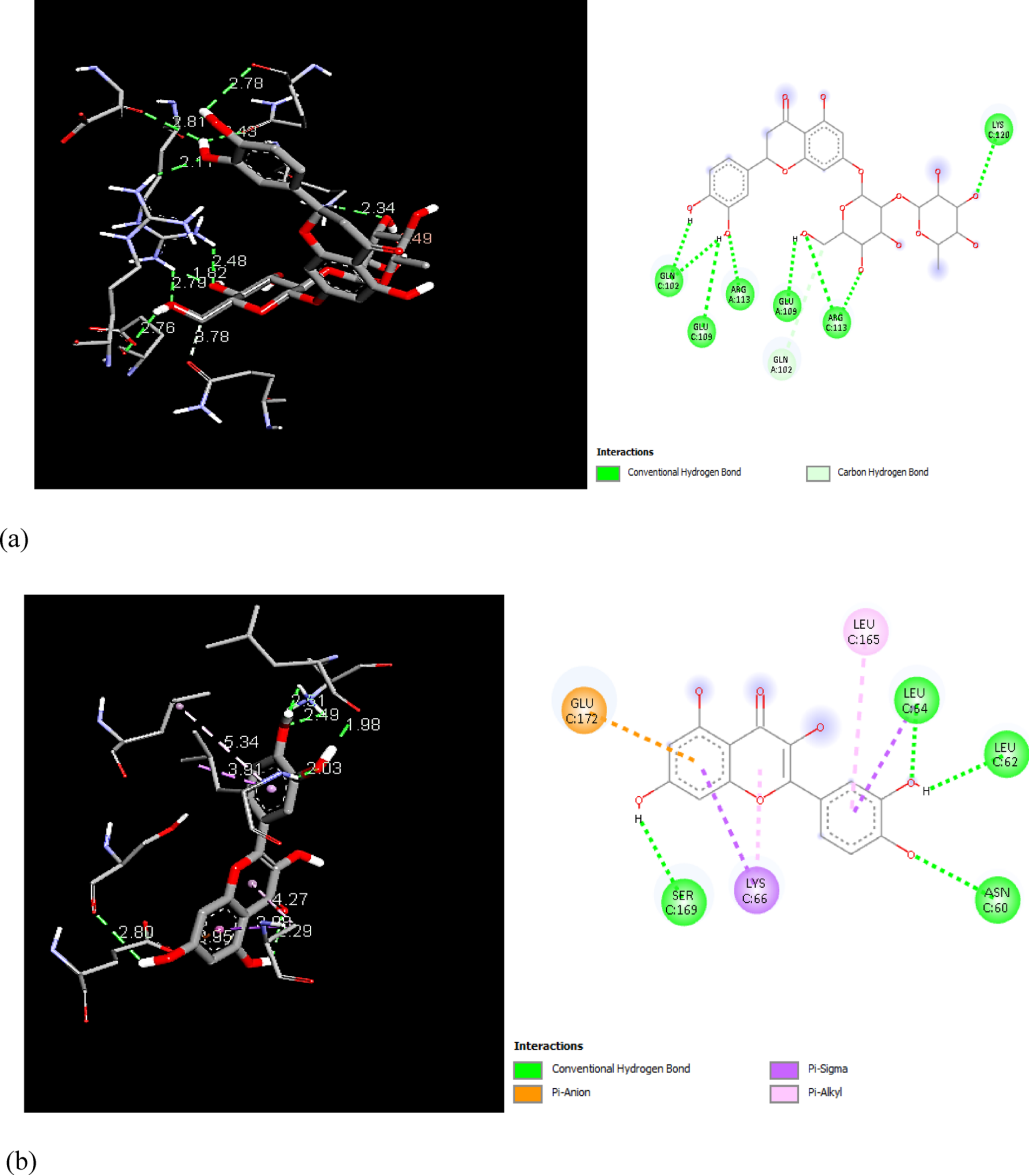

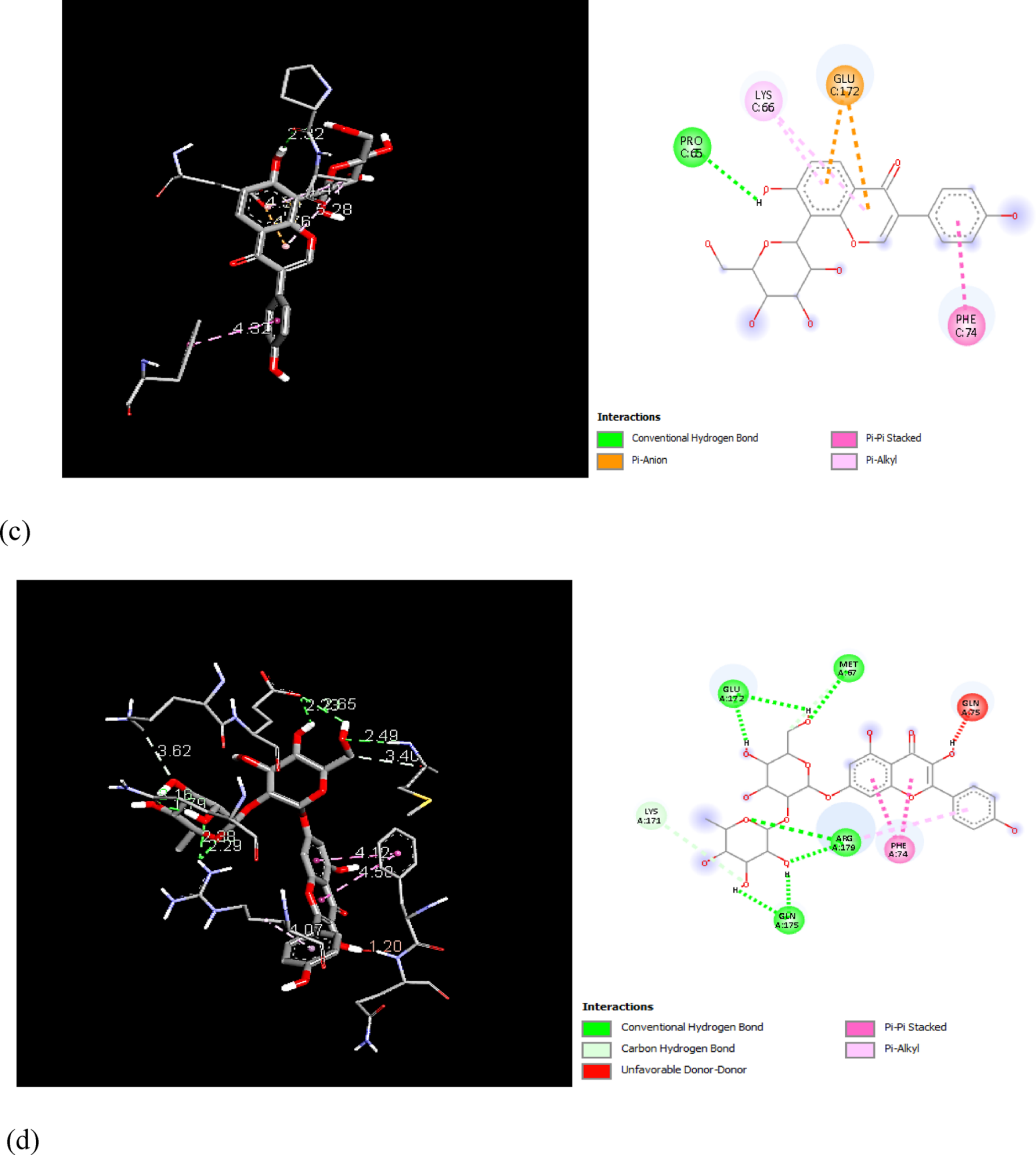
3D and 2D interactions of the isolated compounds from *Convolvulus oxyphyllus* Boiss within interleukin-6 active site. (**a**) Eeriodictyol-7-O-neohesperidoside, (**b**) Quercetin, (**c**) Daidzein-8-C-glucoside, and (**d**) Kaempferol-7-neohesperidoside.

In order to examine the possible compound/enzyme-binding relationships, compounds quercetin, daidzein-8-C-glucoside, kaempferol-7-neohesperidoside and Eeriodictyol-7-O-neohesperidosid were docked into the active site of cycloxygenae-2 (PDB code 8ET0) (Fig. 5) and interleukin-6 (PDB code 4N19) (Fig. 6). Table 3 and Figs. 7, 8a–d, 9 and 10a–d emphasize 3D and 2D interactions representations of the docked poses. The energies showed by ligands for cycloxygenae-2 range from (-10.2 to -9.6 Kcal/mol); the highest binding affinity Eeriodictyol-7-O-neohesperidoside (-10.2 Kcal/mol) with interacting amino acid residues (CYS15, PRO122, CYS4, GLN430, VAL124, GLY93, GLN296, ALA125, SER17, MET16 and GLY104); followed by Quercetin (-9.9 Kcal/mol) with interacting amino acid residues (THR175, HIS355, HIS176, ASP101 and HIS357) then Daidzein-8-C-glucoside and kaempferol-7-neohesperidoside (-9.7 Kcal/mol and − 9.6 Kcal/mol, respectively). Concerning the energies for interleukin-6 (-7.4 to -6.9 Kcal/mol); the highest binding affinity kaempferol-7-neohesperidoside (-7.4 Kcal/mol) with interacting amino acid residues such as (MET67, GLU172, LYS171, GLN175, ARG179 and PHE74); then Eeriodictyol-7-O-neohesperidoside (-7.2 Kcal/mol) with interacting amino acid residues (LYS120, ARG113, GLU109, ARG113, GLU109 and GLN102) followed by both Quercetin and Daidzein-8-C-glucoside (-6.9 Kcal/mol). However, the standard Celecoxib binding affinity was (-8.7 Kcal/mol and − 6.8 Kcal/mol) for both enzymes, respectively; which lower than that exerted by the tested compounds.

The analysis of these results suggests that the compounds may have potential as anti-inflammatory agents, warranting further in vitro and in vivo studies to validate their activity and facilitate their future development. While the docking results provide insights into possible interactions with inflammatory targets, they predict binding potential rather than confirm actual enzymatic inhibition, and experimental validation (e.g., COX-2 inhibition assays and cytokine or cell-based assays) is required to substantiate these findings. In the context of this study, docking to IL-6 serves as a mechanistic hypothesis generator, helping to prioritize bioactive compounds from *Convolvulus oxyphyllus* for future experimental investigation, while emphasizing that conclusions regarding IL-6 inhibition must remain tentative until validated.

**Table 3:** Docking results of the isolated compounds from *Convolvulus oxyphyllus Boiss* and Celecoxib within the active site of cycloxygenae-2 and interleukin-6 enzymes.

	Binding affinity (Kcal/mol)		COX2			IL-6		
	COX2	IL-6	Amino acids	Interacting groups	Type of interaction	Amino acids	Interacting groups	Type of interaction
Celecoxib	-8.7	-6.8	GLN512	NH	Hydrogen bond	GLU99	NH	Hydrogen bond
			TYR91	Aryl	pi-pi-stacked	GLN116	O	Hydrogen bond
			ARG29	CH_3_	Alkyl	LYS120	H, aryl	Hydrogen bond, pi-cation
			PRO511	Aryl	Alkyl	GLU95	H	Hydrogen bond
			GLN341	CH_3_	Hydrogen bond	LEU92	CH_3_	Halogen (fluorine)
						PRO139	CH_3_	C-Hydrogen bond
			SER95	CH_3_	Halogen (fluorine)	VAL96	Aryl	Pi-alkyl
						ALA145	CH_3_	Pi-alkyl
						LEU148	CH_3_	Pi-alkyl
Eeriodictyol-7-O-neohesperidoside	-10.2	-7.2	CYS15	Aryl	Pi-alkyl	LYS120	O	Hydrogen bond
			PRO122	Aryl	Pi-alkyl	ARG113	O, O	Hydrogen bond
			CYS4	Aryl	Pi-alkyl	GLU109	H	Hydrogen bond
			GLN430	O	Hydrogen bond	ARG113	O	Hydrogen bond
			VAL124	CH_3_	C-Hydrogen bond	GLU109	H	Hydrogen bond
			GLY93	O	Hydrogen bond	GLN102	H, H, CH_3_	Hydrogen bond C-Hydrogen bond
			GLN296	H, H	Hydrogen bond			
			ALA125	Aryl	Pi-alkyl			
			SER17	O	Hydrogen bond			
			MET16	O	C-Hydrogen bond			
			GLY104	CH_3_	C-Hydrogen bond			
Quercetin	-9.9	-6.9	THR175	O	Hydrogen bond	ASN60	O	Hydrogen bond
			HIS355	Aryl	Pi-Cation	LEU62	H	Hydrogen bond
			HIS176	Aryl, O	pi-pi-stacked, C-Hydrogen bond	LEU64	O, Aryl	Hydrogen bond, Pi-sigma
			ASP101	Aryl	Pi-anion	LEU165	Aryl	Pi-alky
			HIS357	O, O	Hydrogen bond, C-Hydrogen bond	GLU172	Aryl	Pi-anion
						SER169	H	Hydrogen bond
						LYS66	Aryl, Aryl	Pi-sigma, Pi-alky
Daidzein-8-C-glucoside	-9.7	-6.9	CYS4	H	Hydrogen bond	GLU172	Aryl	Pi-anion
			CYS15	H, H	Hydrogen bond	LYS66	Aryl	Pi-alkyl
			ASP126	H, aryl	Hydrogen bond, pi-anion	PRO65	H	Hydrogen bond
			VAL124	Aryl	Amide-pi stacked	PHE74	Aryl	Pi-pi-stacked
			ALA125	aryl	Pi-alkyl			
kaempferol-7-neohesperidoside	-9.6	-7.4	GLY194	H	Hydrogen bond	MET67	O	Hydrogen bond
			ASN344	H, O	Hydrogen bond	GLU172	H, H	Hydrogen bond
			GLN343	H	Hydrogen bond	LYS171	O	C-Hydrogen bond
			GLU205	Aryl	Pi-anion	GLN175	H, H	Hydrogen bond
			LEU114	Aryl	Pi-alkyl	ARG179	O, O. Aryl	Hydrogen bond Pi-alkyl
			SER112	Aryl	Pi-sigma	PHE74	Aryl	Pi-pi-stacked
			TRP108	Aryl	Pi-pi-T-shaped			
			ARG302	O	Hydrogen bond			

## Conclusion

This study demonstrates that *C. oxyphyllus* extracts, particularly in selenium nanoparticle (Se-NP) form, effectively downregulate IL-6 and COX-2 transcription in LPS-stimulated RAW264.7 macrophages, highlighting their potential as anti-inflammatory agents. The nanoformulation likely enhances the stability, bioavailability, and cellular uptake of the plant’s bioactives, contributing to the observed efficacy. Molecular docking further supports these findings by predicting favorable interactions of key flavonoid glycosides—eriodictyol-7-O-neohesperidoside with COX-2 (− 10.2 kcal/mol) and kaempferol-7-neohesperidoside with IL-6 (− 7.4 kcal/mol)—with critical residues of the inflammatory targets.

The combined downregulation of IL-6 and COX-2, alongside these predicted ligand–target interactions, suggests that *C. oxyphyllus* extracts in nanoform may offer a natural alternative or adjunct to conventional NSAID therapy, potentially driven by their flavonoid content. However, as docking results are predictive, further experimental validation is essential. Future studies should include protein-level assays (ELISA or Western blot), dose–response and cytotoxicity evaluations, and in vivo assessments to fully establish their therapeutic potential and mechanism of action.

## Data Availability

The datasets used and/or analyzed during the current study available from the corresponding author (R.A.L.) on reasonable request.
